# Primary Bacterial Ventriculitis caused by *Staphylococcus warneri*

**DOI:** 10.1590/0037-8682-0631-2022

**Published:** 2023-03-06

**Authors:** Fatma Şimşek, Recep Yevgi, Ahmet Yalçın

**Affiliations:** 1 Ataturk University, Faculty of Medicine, Department of Neurology, Erzurum, Turkey.; 2 Ataturk University, Faculty of Medicine, Department of Radiology, Erzurum, Turkey.

**FIGURE 1:**
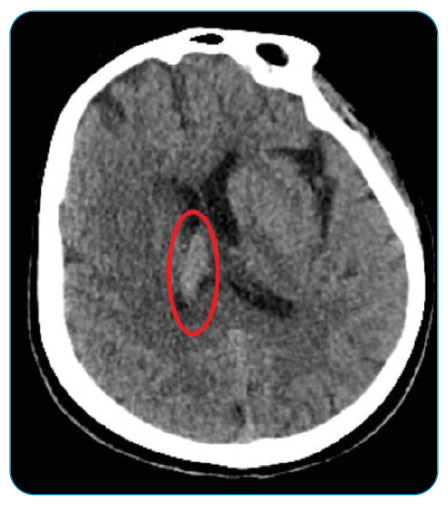
CT of the brain showing a hyperdense area is observed in the right lateral ventricle.

**FIGURE 2:**
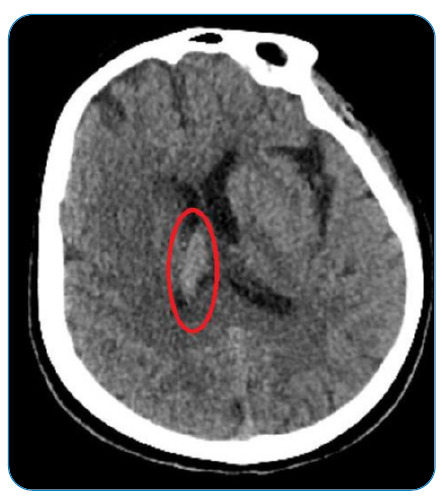
MRI of the brain showing more prominent bilaterally hyperintense areas adjacent to the right lateral ventricle in the FLAIR sequence. This was evaluated as being compatible with ventriculitis.

**FIGURE 3:**
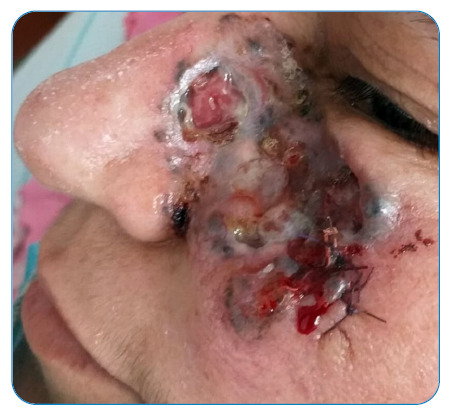
Clinical photograph showing an ulcerated and hyperpigmented wound with discharge in the left nose-cheek region.

A 51-year-old female patient was evaluated at Atatürk University Hospital for headache and drowsiness. She had no fever, neck stiffness, or motor deficits. A hyperdense area in the right lateral ventricle was observed on brain computed tomography (CT) (Figure 1). More prominent bilateral hyperintense areas adjacent to the right lateral ventricle were observed in the FLAIR sequence on magnetic resonance imaging (MRI). Based on the imaging, a diagnosis of ventriculitis was proposed (Figure 2). A lumbar puncture (LP) was performed. Cerebrospinal fluid (CSF) demonstrated a xanthochromic appearance. The glycoprotein level of the CSF was 60 mg/dL, and the glucose level was 62 mg/dL (simultaneous blood glucose level was 105 mg/dL). Direct examination revealed 4 lymphocytes. Ceftriaxone (1 g, 2 × 2) was initiated. *Staphylococcus warneri* was detected in the CSF. The patient had an infected, ulcerated, and hyperpigmented wound in the left nose-cheek region, which was said to have been present for 2-3 years (Figure 3). The histopathological examination was compatible with squamous cell carcinoma. No other source of infection was found. The cause of ventriculitis was thought to be an infected wound and the complex lymphatic drainage of the head region. The patient did not undergo a repeat LP. Clinical improvement was evident after 21 days of antibiotic treatment. *Staphylococcus warneri* constitutes <1% of the staphylococcal flora of the skin^1^. Although ventriculitis mostly develops due to external ventricular drains, cases of primary bacterial ventriculitis have rarely been reported^2^. Importantly, *Staphylococcus warneri* rarely causes primary ventriculitis.
