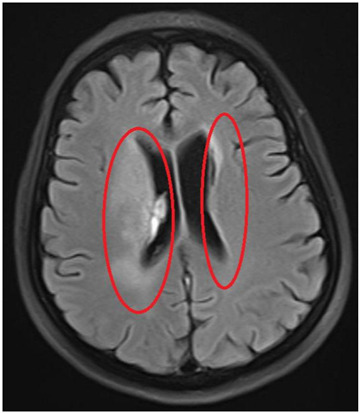# Erratum

**DOI:** 10.1590/0037-8682-0631b-2022

**Published:** 2023-03-27

**Revista da Sociedade Brasileira de Medicina Tropical/Journal of the Brazilian Society of Tropical Medicine**

**Title:** Primary Bacterial Ventriculitis caused by *Staphylococcus warneri*

**56: (e0631-2022) 2023 - Page: 1/3 - doi:** https://doi.org/10.1590/0037-8682-0631-2022

**FIGURE 2:**

**Figure:**
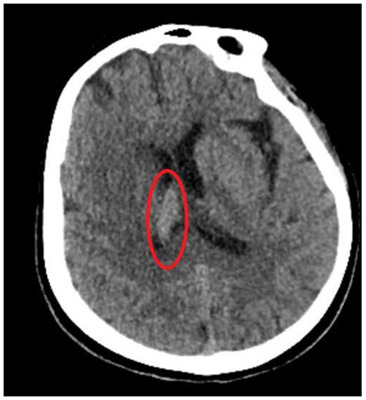

**Should read:**

**Figure:**